# Investigations on antibody binding to a micro-cantilever coated with a BAM pesticide residue

**DOI:** 10.1186/1556-276X-6-386

**Published:** 2011-05-16

**Authors:** Michael Bache, Rafael Taboryski, Silvan Schmid, Jens Aamand, Mogens Havsteen Jakobsen

**Affiliations:** 1 Department of Micro- and Nanotechnology, Technical University of Denmark, DTU Nanotech, Building 345 East, 2800 Kongens Lyngby, Denmark; 2 Department of Geochemistry, Geological Survey of Denmark and Greenland (GEUS), Øster voldgade 10, DK-1350 Copenhagen K, Denmark

## Abstract

The attachment of an antibody to an antigen-coated cantilever has been investigated by repeated experiments, using a cantilever-based detection system by Cantion A/S. The stress induced by the binding of a pesticide residue BAM (2,6 dichlorobenzamide) immobilized on a cantilever surface to anti-BAM antibody is measured using the CantiLab4^© ^system from Cantion A/S with four gold-coated cantilevers and piezo resistive readout. The detection mechanism is in principle label-free, but fluorescent-marked antibodies have been used to subsequently verify the binding on the cantilever surface. The bending and increase in mass of each cantilever has also been investigated using a light interferometer and a Doppler Vibrometer. The system has been analyzed during repeated measurements to investigate whether the CantiLab4^© ^system is a suited platform for a pesticide assay system.

## Introduction

During the last 10 years an increasing number of water wells have been polluted by pesticides or its break down products. BAM is among the most frequent found pesticide residues in European groundwater. As pesticide analysis of drinking water is currently being done by laboratory analysis, an in-line sensor will therefore be beneficial for water quality monitoring. Cantilever-based assays for pesticide detection has been reported [1,2], but few description of repeated measurements using cantilever-based detection systems are available. As a central principle of a possible cantilever-based competitive assay, we have tested the binding of a BAM antibody to a cantilever surface passive coated with a BAM ovalbumine conjugate. In a working assay, the BAM molecules in a water sample would compete with BAM attached to a cantilever surface for the binding to anti-BAM monoclonal antibodies, similar to a BAM ELISA described by Bruun et al [3]. The binding of anti-BAM antibodies to the surface of the cantilever will change the surface stress, causing bending of the cantilever. The bending is then detected by a change in resistance of the imbedded piezoelectric layer in the cantilever [4-6]. To investigate whether the system is suited as a transducer for a pesticide bio-assay, the variance of the cantilever bending signal during 10 antibody binding experiments was analyzed. The mechanical properties of the cantilevers were also monitored by measuring the cantilever bending profile, cantilever mass/stiffness, and antibody fluorescent signal. This was repeated on the clean cantilevers, after the cantilevers were functionalization with antigens, and after the antibody was added.

## Materials and methods

A cantilever system CantiChip4^® ^from NanoNord/Cantion A/S was chosen for the assay. The bending of the cantilever causes a proportional change in voltage between the piezo layer in the cantilever and a fixed resistor embedded in the chip measured via a Wheatstone bridge setup. The system consists of four silicon-based cantilevers with integrated piezo resistive readout. All four cantilevers are 120 μm length × 50 μm width × 0.45 μm thickness, coated with a 40-nm gold layer, electrically grounded, and flip chip bonded to a contact pad. The CantiChip4^® ^is inserted in the CantiLab4^© ^that converts the voltage signal to proprietary recording software [7]. The functionalization of each cantilever was done using a micro-spotter from Cantion A/S with a piezo electric controlled pin head (GESIM Sub-Micro liter Piezoelectric Dispenser A010-006 SPIP) in a *xyz *stage setup monitored via a camera and a PC interface. A 2,6 dichlorobenzamide hapten (BAM hapten EQ0031) and ovalbumine conjugate was synthesized following Bruun et al [3]. The BAM ovalbumine conjugate was dialyzed 3× in 1× PBS buffer, and diluted to 0.75 mg/ml of ovalbumine in 1× PBS. The BAM-ovalbumine conjugate was determined to contain 5 U BAM/ovalbumine via a UV-Visual spectrophotometer method and was tested positive for BAM via an ELISA [3].

**Figure 1:**
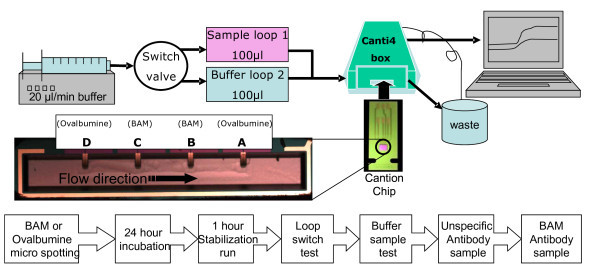
**Experimental setup overview.** (Above) A schematic overview of the fluidic setup; (Below) Flowchart of the BAM assay on the CantiChip4^® ^system .

On an inspected, tested, and clean CantiChip4^®^, three drops of 0.75 mg/ml BAM-ovalbumine in 1× PBS buffer solution was micro-spotted on cantilever B and C, using a tip voltage of 100 V and pulse width of 20 V. Cantilever A and D was used as reference and was equally micro-spotted with a 1 mg/ml ovalbumine in 1× PBS buffer solution (Figure 1). The chip was incubated overnight in a humidity chamber. A functionalized chip was inserted in the CantiLab4^© ^connected to a fluidic system that consisted of a syringe pump and an 8 channel switchbox. The system was allowed to heat up and stabilize with a continuous flow of 20 μl/min of 1× PBS 0,05% Tween 20 pH 7.4 buffer, for approximately 1 h while a base line was recorded. The experiment consisted of a four-step protocol to minimize false signal sources. The system was first tested against any signal induced by loop switching, second against signal due to buffer injected as a sample. To test for any unspecific antibody attachment signal, a sample of 100 μl of 0.1 mg/ml unspecific mouse Immunoglobulin G (Sigma-Aldrich reagent grade I5381-1 mg, lot.nr.025K7580) Cy5 labeled (Amersham Cy5 Dye™ Antibody monofunctional Labeling Kit) was injected. Following a 5-min buffer flow, finally an injection of 100 μl of the 0.1 mg/ml BAM antibody (Statens Serum Institut, HYB 273-01, Batch nr.03102P01/071008) labeled with Cy3 fluorochrome (Amersham Cy3 Dye™ Antibody mono functional Labeling Kit) was done. Both antibodies were diluted in 1× PBS 0.05%. Tween 20. After the experiment, the Cantion chip was removed from the fluidic chamber and briefly rinsed in Milli-Q water to remove PBS salts. Each test and injection phase of the assay was recorded for 2000 s. The total experiment lasted typically 31/2 h, depending on quality of the output signal and the stabilization period.

In order to verify the binding of antibodies to the cantilever surface and control for unspecific binding, a set of fluorescent pictures of Cy5 and Cy3 signal were taken after spotting and antibody attachment. An optical surface profilometer (Polytech TMS-100), based on light interference, was used to analyze the absolute bending of the cantilevers on five experiments. To analyze the mass/stiffness values, a laser-based vibrometer with a piezo actuator (Doppler Vibrometer Polytech MSA 500) was used on eight experiments [8]. All chemicals used in the assay were purchased via Sigma Aldrich Denmark; only new glassware was used and rinsed in Milli-Q water to avoid any unwanted effect from surfactants.

## Results and discussion

**Figure 2:**
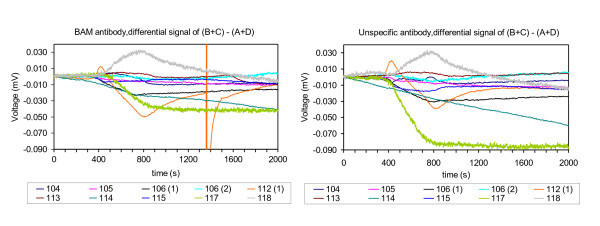
**Comparison of bending signals from 10 experiments**. The differential signal between the two signal BAM-coated and two reference ovalbumine-coated cantilevers is shown. Plotted as signal (mV) of (B + C) - (A + D) as a function of time (s) during the addition of BAM antibody (left) and unspecific antibody (right).

Twenty chips were selected for the BAM assay based on signal stability while running in air mode and a buffer flow. Of 20 experiments, only ten gave a signal when adding BAM antibody (with five experiments giving a differential signal above 0.01 mV) (Figure 2). Seven experiments gave no differential signal, and three chips were discarded after functionalization, due to too high initial voltage difference between the cantilevers. A signal from the addition of specific BAM antibody, as well as from the addition of unspecific antibody appeared on all 10 successful experiments. The differential signals show a very diverse and distinct signal profile in between experiments, but has a similar signal profile between the specific and the unspecific antibody on each experiment (Figure 2).

**Figure 3:**
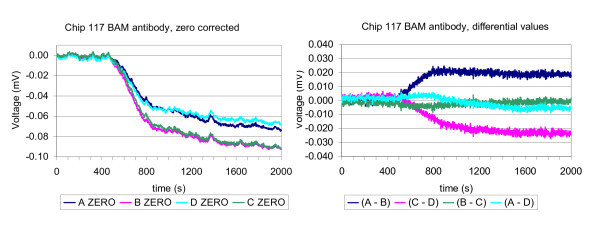
**Bending signal example**. (Left) An example of a bending signal from cantilever A, B, C, D normalized to zero at *t *= 0 under the addition of BAM antibody; (Right) The differential values of A (signal) - B(reference), C(reference) - D(signal), B(signal) - C(signal), and A(reference) - D(reference) . The antibody is added at *t *= 200 and enters the cantilever chamber at *t *= 500, gradually causing a lasting differential signal of approximately 0.02 mV for the specific antibody.

Baseline noise was typically in the range of 0.004 to 0.002 mV. (Figure 3, left). As the absolute bending signal were not suited to evaluate the experiment, the differential values of A(signal) - B(reference), C(reference) - D(signal), B(signal) - C(signal), and A(reference) - D(reference) were plotted, a signal example from chip 117 is seen in Figure 3.

**Figure 4:**
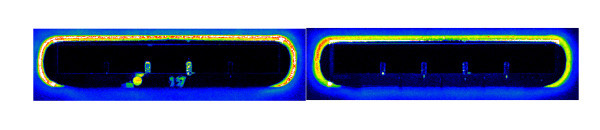
**Fluorescent antibody signal. **(Left) An example of a clear fluorescent signal (ex. 550 nm, em. 570 nm) of Cy3 marked anti-BAM to cantilever B and C and none on A and D; (Right) Fluorescent picture of Cy5 signal (ex. 650 nm, em. 670 nm) from chip 113, showing a low background signal of Cy5 marked unspecific mouse antibody .

Although the functionalization of the cantilevers (using micro-spotting and passive adsorption to the gold layer) is the source of some of the variations obtained in the differential values, it cannot fully explain the large variations of the signal. As observed by Dauksaite et al. [5], almost all experiments had a signal drift effect during the experiment varying from a few μV to several mV/h, possibly caused by a variance in the resistance of the internal resistors of the Wheatstone bridge on the chip. Another observation was a battery effect in the fluidic system causing a 0.1-1 mV signal change. To avoid this battery effect between the chip and fluidic system, the waste bottle was connected to the CantiLab4^© ^electronic box with a gold wire (Figure 1). A weak loop switch effect of 0.01-0.004 mV was also observed when switching the fluidic loop, this was probably caused by minute changes of pH or salinity in the sample buffer resting in the sample coil. The effect immediately disappeared after one loop switch and was avoided using a continuous buffer flow. As the antibody-antigen binding is mainly controlled by electrostatic forces [9] and the bending signal is found to be very sensitive to minute changes in pH, salinity, and temperature gradients [10], we wanted to investigate whether the unspecific antibody signal was just caused by the relatively high antibody concentration (0.1 mg/ml). 2 experiments with 1/10 (0.01 mg/ml) antibody were done, but unfortunately these showed no signal when the antibody was added (data not shown). The BAM antibody was marked with Cy3 fluorochrome and the unspecific mouse IgG antibody with Cy5 to assure that the BAM antibody was attached to cantilevers B and C and no unspecific antibody was attached. All 10 experiments had a similar Cy3 signal from BAM antibody on cantilever B and C and little or no Cy3 signal on cantilever A and D. No significant amount of Cy5 marked unspecific antibody signal was found on any cantilever after the experiment. A typical Cy3 and Cy5 fluorescent signal is seen in Figure 4.

**Figure 5:**
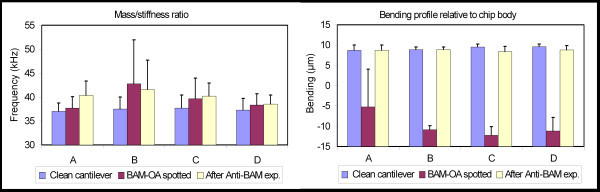
**Cantilever resonance frequency and bending.** (Left) Mass/stiffness ratios of each of the four cantilevers divided in three groups: clean chip, after functionalization by micro-spotting, and after the addition of BAM antibody . The values are an average of eight experiments (104, 108, 112, 116, 117, 118, 119, and 120). (Right) Average bending values (μm) of cantilever tip relative to the chip body surface. Values are averages obtained from five experiments (116, 117, 118, 119, and 120).

The deflection values showed a clear bending of all cantilevers after the functionalization step (Figure 5, right). This was probably caused by salt deposits from the PBS buffer used in the micro-spotting of BAM-ovalbumine conjugate and ovalbumine. The cantilevers returned to their initial state after the experiment, probably caused by the removal of these deposited salts from the functionalization step. A large variation on the resonance frequency could explain the diverse signal variations obtained. The cantilevers showed a slight increase in variation of the resonance frequency after the functionalization step; but no significant difference could be seen after the experiment was performed (Figure 5, left).

Although three chips were discarded during the 20 experiments, the Cantion chips were able to perform a continuous voltage readout lasting several days. The Cantion chips could also be re-used following a rinsing protocol. This opens up the possibility of regeneration of the surface chemistry by repeated assays, using only one sensor in an automated system. However, the system was not found suitable as a platform for a pesticide bio-assay in its current form, as the quality of the differential signal was not repeatable. The fluorescent pictures of anti-BAM showed repeated attachment only to the BAM functionalized cantilever surfaces, and no binding of unspecific Cy5 marked antibody. The signal variation is therefore unlikely to be caused only by variations in the cantilever functionalization step. The variations are more likely caused by minute changes in buffer pH, temperature, and salinity, as this affects the electromagnetic field surrounding the cantilever (caused by the 2.5 V tension in the cantilever piezo layer). The very large antibody concentrations needed to obtain a differential signal on the system is believed to be the cause of the signal from the unspecific antibody as it interacted with the cantilever surface, but this could not be proved in the experiments.

## Competing interests

The authors declare that they have no competing interests.

## Authors' contributions

MHJ conceived the study. RT assisted in the design of the study. JA provided Anti-BAM antibody and BAM hapten EQ0031. SS performed the cantilever bending and resonance frequency measurements. MB performed the experiments, designed the study and wrote the manuscript. All authors read and approved the final manuscript.
